# Breakfast Consumption Habits at Age 6 and Cognitive Ability at Age 12: A Longitudinal Cohort Study

**DOI:** 10.3390/nu13062080

**Published:** 2021-06-17

**Authors:** Jianghong Liu, Lezhou Wu, Phoebe Um, Jessica Wang, Tanja V. E. Kral, Alexandra Hanlon, Zumin Shi

**Affiliations:** 1 Department of Family and Community Health, University of Pennsylvania, Philadelphia, PA 19104, USA; phoebeum@sas.upenn.edu (P.U.); jeswang@sas.upenn.edu (J.W.); tkral@nursing.upenn.edu (T.V.E.K.); 2 Department of Biomedical and Health Informatics, Children’s Hospital of Philadelphia, Philadelphia, PA 19104, USA; wul5@chop.edu; 3 Center for Biostatistics and Health Data Science, Virginia Polytechnic Institute and State University, Blacksburg, VA 24061, USA; alhanlon@vt.edu; 4 Human Nutrition Department, College of Health Sciences, QU Health, Qatar University, Doha 2713, Qatar; zumin@qu.edu.qa

**Keywords:** breakfast, breakfast composition, cognition, IQ, academic achievement

## Abstract

This study aimed to assess the relationship between breakfast composition and long-term regular breakfast consumption and cognitive function. Participants included 835 children from the China Jintan Cohort Study for the cross-sectional study and 511 children for the longitudinal study. Breakfast consumption was assessed at ages 6 and 12 through parental and self-administered questionnaires. Cognitive ability was measured as a composition of IQ at age 6 and 12 and academic achievement at age 12, which were assessed by the Chinese versions of the Wechsler Intelligence Scales and standardized school reports, respectively. Multivariable general linear and mixed models were used to evaluate the relationships between breakfast consumption, breakfast composition and cognitive performance. In the longitudinal analyses, 94.7% of participants consumed breakfast ≥ 4 days per week. Controlling for nine covariates, multivariate mixed models reported that compared to infrequent breakfast consumption, regular breakfast intake was associated with an increase of 5.54 points for verbal and 4.35 points for full IQ scores (*p* < 0.05). In our cross-sectional analyses at age 12, consuming grain/rice or meat/egg 6–7 days per week was significantly associated with higher verbal, performance, and full-scale IQs, by 3.56, 3.69, and 4.56 points, respectively (*p* < 0.05), compared with consuming grain/rice 0–2 days per week. Regular meat/egg consumption appeared to facilitate academic achievement (mean difference = 0.232, *p* = 0.043). No association was found between fruit/vegetable and dairy consumption and cognitive ability. In this 6-year longitudinal study, regular breakfast habits are associated with higher IQ. Frequent grain/rice and meat/egg consumption during breakfast may be linked with improved cognitive function in youth.

## 1. Introduction

Increasing research has demonstrated associations between breakfast consumption and numerous health benefits, including higher overall diet quality, lower cholesterol, lower rates of obesity and cognitive performance [1,2,3]. Given disparities seen between socioeconomic status (SES) and breakfast consumption, with lower SES being linked to increased breakfast skipping [4,5], understanding the nutritional and cognitive benefits of breakfast is important in the development of interventions aimed at improving health and academic outcomes of low-SES children. Previously, we reported that regular breakfast consumption is associated with increased intelligence quotient (IQ) in kindergarten children [3]. Since our report, several studies examining the relationship between breakfast and cognition have published mixed findings [6,7,8], demonstrating a need for improved methodology and further evaluation of breakfast composition to evaluate potential nutritional corollaries to observed outcomes. First, studies on breakfast consumption and cognitive function have largely been cross-sectional, which by nature, eliminates the author’s ability to determine the directionality of the relationship. Furthermore, cross-sectional studies demonstrated contradicting findings and have been inconclusive in nature. Some found that breakfast consumption improved cognitive performance compared to breakfast omission in children and adolescents [6,7], while others found little or no effect between breakfast consumption and cognition [9,10]. These mixed findings suggest that methodological limitations as well as confounding variables may contribute to the lack of consistent findings. It remains unclear if cognitive differences between frequent breakfast consumers and infrequent breakfast consumers decreases, increases, or remains the same over time. Furthermore, few studies have examined both IQ and school performance, the latter being more likely to demonstrate a longitudinal assessment of student’s cognitive function.

Secondly, if there is an effect of breakfast consumption on cognition, the question remains regarding the relationship between the contents of varying breakfasts and cognition, if one is to exist. Evidence thus far on the association between breakfast composition and cognitive functioning has been inconclusive and limited [6,11,12,13]. Interventional studies have suggested that lower glycemic index (GI) breakfasts may be linked to better cognition when compared with higher GI breakfasts [6]. However, these studies have primarily investigated western samples, in which breakfast commonly includes breads and cereals in combination with milk, sweet and fatty spreads (i.e., butter, cream cheese), fruit, juice, yogurt, and cheese with limited consumption of meat and soy products [6,12]. Furthermore, studies that use continuous blood glucose monitoring have failed to demonstrate a relationship between postprandial glycemic responses and cognitive performance [11]. Taken together, these studies have demonstrated unclear utility of the use of GI and glycemic load to analyze the effect of breakfast composition, thus necessitating the use of novel ways to assess the effects of specific breakfast components.

Our study aims to expand current research on the relationship between breakfast and cognition by addressing two gaps in the literature. Using a unique two-wave data set, we aim to perform both longitudinal and cross-sectional analyses in our original China Jintan Cohort to study whether consistent breakfast consumption has longitudinal effects on cognitive function and whether the composition of the breakfast impacts cognitive performance. Our first study is a longitudinal analysis that examines whether regular breakfast consumption at age 6 through 12 is associated with higher cognitive function at age 12. Furthermore, our second study is a cross-sectional analysis of breakfast composition and cognitive ability that investigates three crucial questions: (1) the effect of breakfast composition on cognitive ability, (2) the directionality of a putative breakfast composition-cognition relationship, and (3) whether the breakfast composition-cognition relationship extends beyond IQ and also toward applied scholarship (i.e., school performance).

## 2. Materials and Methods

### 2.1. Subjects

The China Jintan Child Cohort Study is a longitudinal study of 1656 Chinese children (55.5% boys), aged 3–5 years at baseline, who were recruited from four preschools in Jintan. Detailed sampling and research procedures of this larger cohort study have been described elsewhere [3,14,15,16]. Of the 1656 original children, 1269 preschool children aged 6 years with complete data for IQ and parent-reported breakfast consumption participated in the first wave of data collection from 2005–2007. The second wave of data collection occurred in 2011–2013, during which the original cohort participants, who now had attended four different elementary schools, completed a self-administered nutrition questionnaire and IQ test. After accounting for those lost to follow-up or had changed schools, 511 children were included in the longitudinal analysis and 835 children were included in the cross-sectional analysis at age 12. Institutional Review Board (# 811114) approval was received from both the ethical committee for research of Jintan Hospital and the University of Pennsylvania. Both breakfast consumption and cognitive function data collection took place at two time points when children were ages 6 and 12.

### 2.2. Measures

#### 2.2.1. Breakfast Consumption at Age 6 and 12

Informed consent and assent were obtained from the parents and children, respectively. Breakfast consumption was assessed at age 6 during the first wave and again at age 12 during the second wave. In the first wave, breakfast consumption habits were measured by asking parents “In a typical week, how often do your children have breakfast? 1 = always (6–7 days a week), 2 = often (4–5 days per week), 3 = sometimes (2–3 days a week), 4 = rarely (0–1 day a week)”.

Breakfast consumption at age 12 was assessed by asking children, “In a typical week, how many days do you have breakfast within an hour after waking up?” with responses ranging from 0 to 7. Furthermore, breakfast composition was assessed by asking “In a typical week, how often do you eat a certain food for your breakfast? (1) never, (2) 1–2 days per week, (3) 3–5 days per week, and (4) almost every day (6–7 days per week)”. The foods assessed included fruit/vegetables, grain/rice, meat/egg, dairy products, and soy products. Because of low frequencies observed for categories 1 (never) and 2 (1–2 days per week), these categories were combined for the analysis.

For the cross-sectional analysis, the frequency of breakfast consumption was categorized into three groups (0–2, 3–5, and 6–7 days per week). For the longitudinal analysis, two breakfast consumption groups (≤3 and >3 days per week) were formed. In addition, we created a 4-category variable of breakfast consumption which combined breakfast intake from both waves. These four categories were: (1) children not regularly consuming breakfast in both waves (“fewer w1 + fewer w2”); (2) children regularly having breakfast in wave 1 but not regularly consuming breakfast in wave 2 (“more w1 + fewer w2”); (3) children not regularly having breakfast in wave 1 but regularly consuming breakfast in wave 2 (“fewer w1 + more w2”); (4) children regularly consuming breakfast in both waves (“more w1 + more w2”).

#### 2.2.2. Cognition (IQ) at Age 6 and 12

Cognition at age 6 was assessed with the Chinese version of the Wechsler Preschool and Primary Scale of Intelligence—Revised (WPPSI–R) [3,17]. The IQ tests were administered to children by certified research assistants who completed the required cognitive assessment training and were blinded from the results. A test-retest reliability is 0.87, and inter-rater reliability is 0.91 in a small sample prior to conducting the large-sample testing. Details of IQ test procedures have been previously reported [3,18]. Verbal subtests were combined to create the Verbal IQ (VIQ) score, and performance subtests were combined to produce the Performance IQ (PIQ) score. Full-Scale IQ (FIQ) score represented a sum of all subtests. Additionally, the reliably and validity of the Chinese version of WPPSI–R in Chinese children has been repeatedly demonstrated [19,20]. In addition, a pilot IQ test conducted before the large-sample testing indicated a satisfactory test-retest and inter-rater reliability, with a correlation coefficient of 0.87 and 0.91, respectively [18].

Cognition at age 12 was assessed using the Wechsler Intelligence Scale for Children-Revised (WISC-R), a standardized test that has demonstrated reliability in Chinese children [21]. Similar to the WPPSI-R, the WISC-R consists of several verbal and non-verbal subtests. The VIQ score was derived from the verbal subtests, the PIQ score was derived from the non-verbal subtests and the FIQ score was a combination of both VIQ and PIQ scores. All IQ tests were administered at Jintan Hospital. To reduce scorer bias, each test received two scores from different individuals. Details of IQ test procedures can be found elsewhere [22,23].

#### 2.2.3. Academic Performance at Age 12

Academic achievement was determined by student grades in three major subjects (Chinese, Math, and English) during the previous semester. These grades were converted to score between one and give, with 1 being “very poor” performance (grade F) and 5 being “very good” performance (grade A). Scores from each subject were combined into a composite score. Further details are included in our previous publication [24,25].

#### 2.2.4. Covariates

Numerous socioeconomic and developmental factors at age 6 were used as covariates, including child sex, parental education (highest degree earned), parental occupation, parental marital status (married/living together versus divorced/separated), maternal age at childbirth, feeding method during infancy (breastfeeding versus formula feeding) [26], breastfeeding duration (months), home location (countryside, town, city), and size of living space per person (m^2^). Parental occupation was collapsed into unemployed, working class, and professional class, with working class referring to physical laborers and professional class denoting occupations requiring a certain skill.

#### 2.2.5. Statistical Analysis

Baseline characteristics of child participants and their families are described using means/standard deviations, or medians/interquartile ranges as appropriate, for continuous outcomes, along with frequencies and percentages for categorical outcomes. Comparisons across groups for breakfast consumption habits at age 12 were performed using chi-square statistics and one-way Analysis of Variance (ANOVA) models, or nonparametric Kruskal Wallis models, for categorical and continuous outcomes, respectively.

For the longitudinal analysis, mixed effect models with robust variances were used to account for correlation within geographic region (preschools and primary schools). Multivariable mixed models were established using a manual covariate selection procedure, such that all variables demonstrating significance at the 0.20 level in bivariate mixed models would be considered in a full model. Then, variables were sequentially removed on the basis of least significance, until only those factors demonstrating significance at the 0.10 level remained. The aforementioned procedure was performed for all three outcome variables (VIQ, PIQ, and FIQ). Based on the literature review and this manual selection procedure, all multivariable models adjusted for child sex, parental education, maternal occupation, feeding method during infancy, and home location. Multivariable generalized linear models (GLM) were used to assess the association between IQ scores measured in wave 2 and the combined breakfast consumption habits. Children who did not consume breakfast regularly in either wave were compared to those who consumed breakfast regularly in both waves, those who consumed breakfast regularly only during wave 1 but not during wave 2, as well as those who consumed breakfast regularly during wave 2 but not during wave 1.

For the cross-sectional analysis at age 12, multivariable GLM analyses were performed to examine the independent dose-response relationship of breakfast frequency with IQ and academic achievement. Multivariable GLM was also used to regress IQ and academic achievement onto each breakfast type (i.e., fruit/vegetables, grain/rice, meat/egg, dairy products, soy products). The resulting models were used to generate least square mean estimates of the outcomes, such that patterns of associations could be visualized using bar charts. GLM was used to regress average academic achievement onto overall breakfast frequency, adjusting for IQ. Exploratory models also included an examination of the interaction or moderating effects of IQ on the association between breakfast frequency and academic achievement. All the statistical analyses were conducted using SAS.

## 3. Results

### 3.1. Sample Characteristics and Breakfast Consumption Profiles

Breakfast consumption habits and baseline characteristics of the study population and their parents are described in Table 1. After the 6-year follow-up period, 511 children were divided into two groups based on breakfast consumption at age 6: participants who reported consuming breakfast at least 4 days per week (*n* = 484 (94.7%)) and those who had breakfast <= 3 days per week (*n* = 27 (5.3%)) children. Most participant characteristics were similar between these two groups (all *p* > 0.03, Table 1), except for father’s education (*p* = 0.011) and maternal age at childbirth (*p* = 0.016). Table 1 also reports that children who regularly ate breakfast at age 6 scored an average of 7.2 points higher on verbal IQ (VIQ) assessments and 5.4 point higher on full IQ (FIQ) assessments than those who did not eat breakfast regularly (all *p* < 0.05), though no significant difference were seen in performance IQ (PIQ) scores (*p* = 0.503).

Of the 835 participants who completed the breakfast questionnaire in 2011–2013 at age 12, children were grouped into three categories based on frequency of breakfast consumption per week: 693 (83.0%) consumed breakfast at least 6 days per week, 110 (13.2%) consumed breakfast 3–5 days per week, and only 32 (3.8%) consumed breakfast less than 3 days per week. Breakfast consumption habits and socio-demographic characteristics of these child participants and their parents are described in Table 1. With the exception of maternal age at childbirth (*p* = 0.023), no significant differences were identified in baseline demographics. Mothers of children who reported consuming breakfast 6–7 days per week were older at childbirth (Table 1). In this cross-sectional analysis, breakfast consumption was positively associated with VIQ (*p* = 0.003), FIQ (*p* = 0.041), and academic achievement (*p* < 0.001). No statistically significant association was found between breakfast consumption and PIQ (*p* = 0.720).

### 3.2. The Longitudinal Association between Breakfast Consumption and Cognition

When compared to less frequent breakfast consumption, regular breakfast consumption was associated with a 5.54-point increased verbal and 4.35-point increased FIQ score (all *p* < 0.05), after controlling for sex, parental education, mother’s occupation, feeding method during infancy, and home location (Table 2). No significant association was found between breakfast consumption habits and PIQ score. Of note, average verbal IQ measured at age 6 was 2.84 points higher than average verbal IQ measured at age 12 (*p* < 0.05), while no comparable changes were observed for PIQ and FIQ scores.

### 3.3. Change in Frequency of Breakfast Consumption and IQ at Age 12

In Table 3, our multivariable generalized linear model demonstrates that children who consumed breakfast regularly during both waves had an average of 19.81-point higher VIQ score (*p* < 0.05) and 12.95-point higher FIQ score (*p* = 0.024) than children who did not consume breakfast regularly during both waves. Additionally, children who regularly ate breakfast at age 12 but not at age 6, as well as those who regularly ate breakfast at age 6 but not at age 12, were more likely to have higher VIQ scores (all *p* < 0.05) than children who did not regularly consume breakfast during both waves. These effects remained significant when all multivariable models were adjusted for covariates, such as sex, father’s education, mother’s education, mother’s occupation, infant feeding methods, and home location. These covariates were identified through bivariate models that regressed outcomes (three IQ scores and academic achievement) on demographic characteristics (Supplementary Table S1).

### 3.4. Dose-Response Relationship between Breakfast Consumption Frequency and Cognitive Ability

Table 4 presents the results of a model examining the dose-response relationship between general breakfast consumption frequency and IQ scores/academic achievement with and without adjustment for covariates. Multivariable analysis indicates that among children 12 years of age, youth who regularly eat breakfast 6–7 days per week were more likely to obtain a significantly higher verbal IQ score (6.760 ± SE; *p* = 0.017) and a higher academic achievement score (0.831 ± SE; *p* < 0.001) compared to youth who rarely eat breakfast (0–2 days per week). The effect of moderate breakfast consumption (i.e., 3–5 days per week) compared to rare breakfast consumption (i.e., <2 days per week) is robust for academic achievement but the association with verbal IQ is less clear. Children who reported moderate breakfast consumption showed significantly higher verbal IQ scores (5.307 ± SE; *p* = 0.092) and academic achievement scores (0.575 ± SE; *p* = 0.007) when compared to youth who reported rare breakfast consumption. No significant relationship was observed between breakfast consumption and performance IQ.

### 3.5. Associations between Breakfast Type and Cognitive Ability

**Figure 1:**
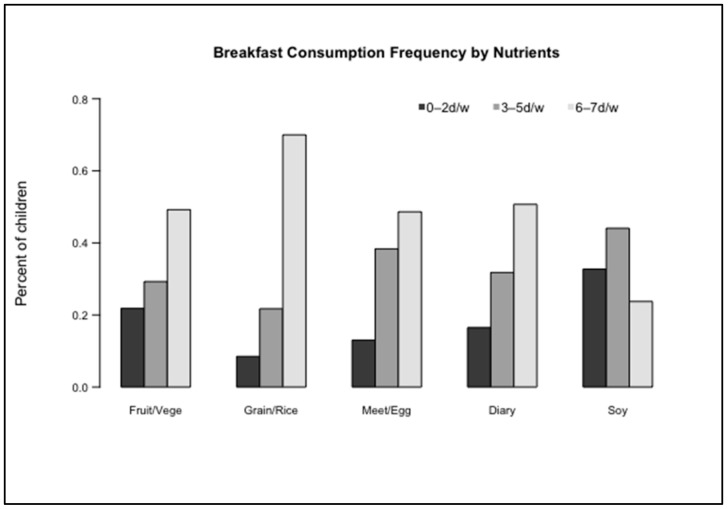
Breakfast Consumption Frequency by Food Type. d/w, days/per week.

**Figure 2:**
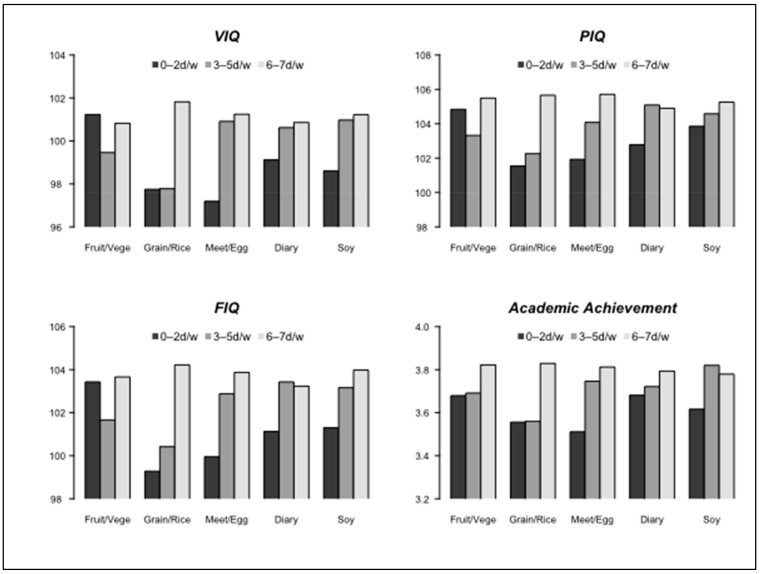
Effect of breakfast food types and consumption frequency on IQ scores/academic achievement. *Note*: The Y-axis denotes estimated marginal means (least square means) of IQ scores and academic achievement using multivariable GLM models.

We further evaluated the effect of different types of foods consumed at breakfast on our four cognitive outcome measures. Figure 1 displays the frequency of types of food consumed at breakfast. Multivariable analysis results shown in Table 5 Section I showed a relationship between frequent consumption of grain/rice or meat/egg during breakfast and higher VIQ, PIQ, and FIQ scores as well as improved academic achievement (all *p* < 0.05), when compared to intake of these foods < 3 days per week. This relationship can be visualized in Figure 2 via marginal means of outcomes as estimated from multivariable GLMs. In Table 5 Section II, intake variables of grain/rice and meat/egg for breakfast were simultaneously forced into the multivariable GLM. Grain/rice intake remained significantly associated with IQ scores indicating that consuming grain/rice food for breakfast on 6–7 days per week increased VIQ, PIQ, and FIQ scores, on average, by 3.562, 3.687, and 4.559 points, respectively (all *p* < 0.05), as compared to grain/rice intake < 3 days per week. Additionally, regular meat/egg consumption at breakfast promoted better academic achievement (6–7 vs. 0–2 days/week, mean score difference = 0.232, *p* = 0.043).

### 3.6. Association between Breakfast Consumption Frequency and Academic Achievement

Finally, Table 6 demonstrates the relationship between breakfast consumption and academic achievement while controlling for VIQ, PIQ, and FIQ. When controlling for IQ and covariates at age 12, children who consumed breakfast on at least three days per week demonstrated better academic achievement than children who consumed breakfast only <3 days per week. No significant interaction between IQ and breakfast consumption was observed.

## 4. Discussion

The findings from this analysis of our second wave data are consistent with previous studies that found associations between regular breakfast consumption and higher IQ scores and higher levels of academic achievement [3,27]. We extend upon these previous findings with a longitudinal analysis that demonstrated an association between long-term breakfast consumption at ages 6 and 12 and higher scores on full and verbal, but not performance, IQ tests. Furthermore, our cross-sectional assessment of breakfast composition and cognitive ability found associations between more frequent breakfast consumption of grain/rice and meat/egg food groups and higher IQ scores and academic achievement. These results remained after controlling for confounding variables, including gender, SES, parental education, maternal occupation, breastfeeding during infancy, and home location.

### 4.1. Longitudinal Effects of Age 6 Breakfast Consumption on Cognition at Age 12

Our study demonstrated that long-term regular breakfast consumption is associated with increased verbal and full IQ scores. Our findings were supported by previous longitudinal studies that studied the effects of school breakfast programs found breakfast consumption to be positively associated with educational outcomes [28], improved memory [29] and improved concentration [30]. As frequent breakfast consumption has been associated with a greater likelihood of meeting daily nutrient guidelines [31], healthier nutritional habits associated with breakfast consumers are likely to play a large role in mediating this relationship between breakfast and cognition. Though not fully elucidated, healthy blood glucose maintenance in children could be a potential mechanism through which breakfast consumption affects cognitive development, particularly in children. Children are known to be more sensitive to glucose supply due to the fact that child brains are relatively larger and display increased activity and growth compared to adult brains [32]. Adequate nutrition during early childhood is crucial for health due to the rapid biological and psychosocial growth that occurs during this period of life. Between birth and age 6, the brain grows to 90% of adult volume [33]. Changes in functional organization, neuronal development and behavior continue through childhood and adolescence as gray and white matter in the brain continue to undergo structural changes of myelination and synaptic pruning [33]. Nutrition is thus a critical component of these changes and previous research has shown that inadequate macro and micronutrient consumption during childhood results in long-term cognitive deficits [34].

The cognitive benefits of social interaction during mealtime could play a large role in the breakfast-cognition relationship. At breakfast time, increased family interaction during a time of psychosocial development may offer cognitive and social benefits. Several studies have reported that frequent family mealtimes are associated with increased literacy [35] and academic achievement [36]. However, this effect is sensitive to the nature of the mealtime interaction; when mealtimes are characterized by openness, responsiveness to children’s questions and social support, children have displayed enhanced language development and academic achievement [37] but this may not be the case for families that consume meals separately. Similarly, for young children, breakfast consumption is often the decision of the parent, rather than the child. As a result, whether or not breakfast is consumed could potentially represent a proxy for parenting style and degree of involvement of the parent. More involved parents may not only give more support physically in terms of nutrition but also emotionally, cognitively, and socially. The presence of supportive, involved parents may also go on to benefit the development of cognitive and verbal skills of children. However, since our relationship between breakfast and consumption persisted despite controlling for SES, the beneficial effect of breakfast on cognitive ability is likely unable to be explained by SES alone, thus suggesting direct nutritional and social benefits associated with breakfast consumption.

### 4.2. Effect of Breakfast Composition on Cognition at Age 12

One possible mechanistic pathway through which breakfast enhances cognition could be through the immediate cognitive impact of breakfast consumption. Our study found that having grain/rice during breakfast 6–7 days per week increased average verbal, performance, and full-scale IQs scores compared with rare grain/rice intake. These results support the findings of previous studies that have suggested that breakfasts rich in complex carbohydrates aid cognitive performance throughout the morning [38]. The role of complex carbohydrates may be mediated by glycemic index (GI) or Glycemic Load (GL), which measure of the rate at which and by how much the breakdown of carbohydrates raises blood glucose levels and the quality and quantity of carbohydrates, respectively. Studies examining the effect of high vs. low GI and GL food on cognition have produced inconsistent results [39,40]. Although several studies have found that low GI, high GL meals predicted better performance in verbal declarative memory tasks and cognitive performance later in the morning [41,42], an Australian cohort of 10- to 12-year-old children found that reducing breakfast Glycemic Load (GL) did not alter satiety or cognition over a 3-h period [11]. However, since our study did not measure specific GI/GL values of the food consumed, we were unable to evaluate the effects of GI/GL differences on cognitive performance.

The role of carbohydrates in restoring low blood glucose levels that occur during sleep [43] offers a potential mechanistic pathway for the observed positive effect of carbohydrate consumption on cognitive performance. As the primary source of energy used by the brain, glucose levels are shown to be associated with enhanced cognitive elements such as memory, reaction time, learning ability and mood [44,45]. Glucose’s widespread effect on several physiological correlates may independently or cooperatively facilitate cognition and affect academic performance. For example, in rats, glucose and epinephrine have been shown to modulate CREB (cAMP-responsive element binding protein) phosphorylation in rats, a process that has been associated with synaptic plasticity, LTP and memory [46].

Our study further demonstrated that regular meat/egg consumption in breakfast was associated with increased academic achievement. Congruent results were found in a study that indicated a positive correlation between academic performance in elementary and high school students and frequent consumption of meats, dairy, and eggs [47]. Proteins positively impact hippocampal function and childhood development of higher cognitive processes [48]. The relationship between protein consumption and hippocampal function was further supported by a study done in rats on protein malnutrition which found that the tasks primarily mediated through hippocampal function were most affected by protein malnutrition [49]. The amino acid tryptophan is commonly consumed through red meat, eggs and poultry and has been shown to benefit sleep [50], mood [51], and cognition [52]. The association between higher sleep quality and cognition is well studied in children and adolescents [53,54,55], suggesting improved sleep quality as possible mediator in the association between protein consumption and academic performance.

Taken together, protein supplementation of carbohydrates appears to be important in improving cognition and academic achievement. A 2012 study in Australian school students found a significant association between habitual breakfast type consisting of carbohydrates and proteins and literacy scores [56]. Similarly, a case study done on a 12-year-old girl indicated that while skipping breakfast and consumption a carbohydrate-rich breakfast of high-sugar cereals increased high-beta brainwave activity associated with anxiety and focus issues, a balanced breakfast of proteins and carbohydrates normalized the high-beta activity [57]. However, it is important to note that although meats and eggs offer nutritional sources of protein, we did not see similar results in children who consumed soy or dairy products, which are also rich sources of protein, which suggests a potential benefit of animal-based protein sources which are often higher in fats. While carbohydrates provide immediate glucose for cognitive enhancement which could explain its effect on singular performance on an IQ test, protein and fats may provide lasting benefits which may be the reason for its significant association with scholastic performance.

### 4.3. Strengths, Limitations, and Future Directions

Previous studies on breakfast consumption have reported inconsistent findings that have made it difficult to elucidate the relationship between breakfast consumption and cognition. While our study has attempted to address several of the methodological challenges of studying the breakfast-cognition relationship, there are several limitations to our study that must be considered when assessing our results. First, it would be more valuable to study a diverse sample population in order to improve the generalizability of the results. Our study performed in a cohort of children in Jintan, China may be influenced by characteristics specific to the diet, culture, and educational system of the city. Second, habitual breakfast consumption was determined through parent and child report, which are subject to limitations of parent or child recall. Furthermore, breakfast consumption was defined as an average of number of days of breakfast intake per week [58] which may in reality differ from week to week. Additionally, our study only assessed the presence or absence of various nutritional components of breakfast but did not assess other factors that could have contributed to the effects of breakfast, such as the quantity of food or the length of breakfast itself, which creates the potential for possible confounding variables. Another potential point of concern is correlation between participant breakfast or cognition habits secondary do unmeasured sibling relationships. However, when this study was conducted, strict birth control policies in mainland China make it very unlikely that a significant portion of the children had siblings between the ages of 3–5. Furthermore, the cross-sectional nature of the association between breakfast type and IQ cannot determine causality, only interrelationships between variables of observation. Thus, future studies should consider longitudinal approaches to investigating this relationship in diverse populations. Lastly, the relationship between nutrition, sleep, and cognition could be intertwined [59]. For example, previous studies have shown that frequent fish consumption in children was associated with better sleep and better cognition [60]. It is possible that children who sleep better have greater time in the morning to eat breakfast, which results in better cognition. However, we did not include sleep in the current study. Future research could take into account breakfast, sleep, and cognition and investigate both mediator and moderator factors among this relationship.

### 4.4. Implications

As these are preliminary findings, it is too early to ascertain a significant association between breakfast and cognition and further research is needed to elucidate the relationship between diet quality and cognition. However, our findings that suggest a potential link between breakfast consumption and composition and cognition emphasize the importance of a healthy eating habit and balanced diet. As children from economically disadvantaged backgrounds often face difficulty in school, this can potentially serve as an intervention point for children at risk of poor developmental and academic outcomes. Being able to provide healthy, balanced breakfasts for students may help enhance both health and cognition in children who may struggle with access to food, thus improving long-term outcomes for underserved children.

It is important to note that breakfast consumption is only a small facet of optimized nutrition. This study contributes to a larger body of evidence supporting the important relationship between nutrition and cognitive development. As cognition and academic performance has downstream effects on school and career-related outcomes, it is in the community’s best interest to understand and promote high-quality nutrition for all students.

## 5. Conclusions

Nutrition has long been recognized as an important factor in the cognitive development of children and adolescents. The findings of this present study suggest a long-term effect of consistent breakfast consumption and that a possible role of breakfast composition as an influence upon the relationship between breakfast and cognition. As academic performance and long-term outcomes are closely related, this study highlights the importance of access to consistent high-quality breakfast especially during youth. Further longitudinal studies and randomized controlled trials are needed to assess the impact of breakfast and nutrition on cognition and academic outcomes.

## Figures and Tables

**Table 1:** Descriptive characteristics by breakfast consumption frequency.

	Longitudinal Analysis			Cross-Sectional Analysis					
	No. of Children (*N* = 511)	Breakfast Consumption at Age 6		*p*-Value	No. of Children (*N* = 835)	Breakfast Consumption at Age 12			*p*-Value
		≤3 d/w (*n* = 27)	≥ 4 d/w (*n* = 484)			0–2 d/w (*n* = 32)	3–5 d/w (*n* = 110)	6–7 d/w (*n*= 693)	
Sex				0.405					0.531
Male	263 (51.5)	16 (59.3)	247 (51.0)		445 (53.3)	20 (62.5)	60 (54.6)	365 (52.7)	
Female	248 (48.5)	11 (40.7)	237 (49.0)		390 (46.7)	12 (37.5)	50 (45.5)	328 (47.3)	
Fathers’ education				0.011					0.072
Less than high school	176 (35.0)	6 (23.1)	170 (35.7)		271 (34.0)	7 (23.3)	39 (37.5)	225 (33.9)	
High school	173 (34.5)	16 (61.5)	157 (33.0)		274 (34.3)	16 (53.3)	40 (38.5)	218 (32.8)	
College or higher	153 (30.5)	4 (15.4)	149 (31.3)		253 (31.7)	7 (23.3)	25 (24.0)	221 (33.3)	
Mothers’ education				0.862					0.322
Less than high school	249 (49.5)	13 (50.0)	236 (49.5)		376 (47.2)	15 (50.0)	58 (55.8)	303 (45.7)	
High school	155 (30.8)	7 (26.9)	148 (31.0)		254 (31.9)	9 (30.0)	31 (29.8)	214 (32.3)	
College or higher	99 (19.7)	6 (23.1)	93 (19.5)		167 (20.9)	6 (20.0)	15(14.4)	146 (22.0)	
Fathers’ occupation				0.646					0.253
Unemployed	21 (4.4)	1 (4.4)	20 (4.4)		28 (3.6)	2 (6.9)	4 (4.0)	22 (3.4)	
Labor Worker	269 (56.0)	15 (65.2)	254 (55.6)		424 (54.8)	18 (62.1)	63 (62.4)	343 (53.3)	
Professional	190 (39.6)	7 (30.4)	183 (40.0)		322 (41.6)	9 (31.0)	34 (33.7)	279 (43.3)	
Mothers’ occupation				0.275					0.528
Unemployed	130 (26.6)	3 (13.1)	127 (27.3)		200 (25.7)	5 (17.9)	32 (31.1)	163 (25.2)	
Worker	216 (44.3)	11 (47.8)	205 (44.1)		324 (41.7)	13 (46.4)	43 (41.8)	268 (41.4)	
Professional	142 (29.1)	9 (39.1)	133 (28.6)		254 (32.7)	10 (35.7)	28 (27.2)	216 (33.4)	
Parents divorced or separated *				0.105					0.197
No	456 (97.6)	22 (91.7)	434 (98.0)		719 (97.2)	27 (93.1)	95 (99.0)	597 (97.1)	
Yes	11 (2.4)	2 (8.3)	9 (2.0)		21 (2.8)	2 (6.9)	1 (1.0)	18 (2.9)	
Maternal age at childbirth	26 (24, 27)	24 (23, 26)	26 (24, 27)	0.016	26 (24, 27)	25(23, 27)	25 (24, 27)	26 (24, 27)	0.023
Infant feeding method				0.457					0.377
Breastfeeding	467 (94.9)	22 (91.7)	445 (95.1)		730 (93.1)	29 (96.7)	93 (90.3)	608 (93.4)	
Formula feeding	25 (5.1)	2 (8.3)	23 (4.9)		54 (6.9)	1 (3.3)	10 (9.7)	43 (6.6)	
Breastfeeding duration (months)	8.8 ± 3.1	9.3 ± 2.4	8.8 ± 3.1	0.463	8.8 ± 3.1	8.9 ± 2.7	8.6 ± 3.5	8.8 ± 2.9	0.797
Home location*				0.527					0.223
Rural	61 (12.1)	5 (19.2)	56 (11.7)		104 (13.0)	7 (22.6)	67 (64.4)	82 (12.4)	
Small Town	84 (16.7)	4 (15.4)	80 (16.8)		128 (16.0)	4 (12.9)	22 (21.2)	102 (15.4)	
City	358 (71.2)	17 (65.4)	341 (71.5)		566 (70.9)	20 (64.5)	67 (64.4)	479 (72.3)	
Living space per person (m^2^)	30.0 (23.5,36.7)	29.0 (24.5, 33.3)	30.0 (23.3, 36.7)	0.896	30.0 (23.3, 38.0)	28.6 (24.0, 46.7)	32.0 (26.7, 40.0)	30.0 (23.3,37.5)	0.331
Siblings				0.279					0.763
No siblings	375 (81.7)	22 (91.7)	353 (81.2)		593 (81.3)	25 (83.3)	74 (78.7)	494 (81.7)	
At least one sibling	84 (18.3)	2 (8.3)	82 (18.8)		136 (18.7)	5 (16.7)	20 (21.3)	111 (18.4)	
Breakfast consumption during wave 2 data collection ^&^				0.013					
≤3 d/w	29 (5.8)	5 (19.2)	24 (5.0)						
≥4 d/w	475 (94.3)	21 (80.8)	454 (95.0)						
IQ during wave 2 data collection									
VIQ	101.6 ± 11.5	94.7 ± 12.2	101.9 ± 11.4	0.002	101.0 ± 12.0	94.5 ± 11.5	99.4 ± 9.8	102.2± 11.6	0.003
PIQ	106.5 ± 12.3	105.0 ± 12.7	106.6 ± 12.2	0.503	105.4 ± 12.0	105.8 ± 12.1	105.3 ± 11.3	106.6 ± 12.3	0.720
FIQ	104.7 ± 12.0	99.6 ± 12.9	105.0 ± 11.9	0.021	103.9 ± 12.9	99.8 ± 11.8	102.6 ± 10.0	105.2 ± 12.1	0.041
Academic achievement					4.0 (3.0, 4.7)	3.0 (2.0, 4.0)	3.7 (3.0, 4.5)	4.0 (3.0, 4.7)	<0.001

Note: Proportions may not add to 100% due to rounding and sum of children by different nominal variables may not add to total due to missing data. Nominal variables were shown as count (column percent). Skewed and normal numeric variables were presented as median (inter-quartile range) and mean +/− standard deviation, respectively. * The Fisher’s exact test was used. ^&^ Numbers do not add to the sum of 511, because seven subjects completed questionnaires but failed to complete information about breakfast consumption. Abbreviation: VIQ, verbal IQ; PIQ, performance IQ; FIQ, full IQ; d/w, days/per week.

**Table 2:** Longitudinal analysis of multivariable mixed model: association between repeatedly measured IQ and breakfast consumption frequency (*n* = 511).

	VIQ		PIQ		FIQ	
	Coefficient (SE)	*p*-Value	Coefficient (SE)	*p*-Value	Coefficient (SE)	*p*-Value
Wave						
First	2.837 (0.63)	<0.001	−1.105 (0.57)	0.054	0.377 (0.55)	0.494
Second	1	Ref	1	Ref	1	Ref
Breakfast consumption						
Always or often	5.537 (1.42)	<0.001	2.195 (1.38)	0.113	4.349 (1.31)	0.001
Sometimes or rarely	1	Ref	1	Ref	1	Ref
Sex						
Female	−2.433 (0.70)	<0.001	−3.048 (0.73)	<0.001	−3.059 (0.68)	<0.001
Male	1	Ref	1	ref	1	ref
Fathers’ education						
College or higher	4.368 (1.08)	<0.001	4.254 (1.12)	<0.001	4.946 (1.05)	<0.001
High school	2.490 (0.90)	0.006	1.428 (0.94)	0.128	2.297 (0.88)	0.009
Less than high school	1	Ref	1	Ref	1	Ref
Mothers’ education						
College or higher	3.534 (1.32)	0.008	2.948 (1.37)	0.032	3.570 (1.29)	0.006
High school	1.128 (0.89)	0.206	2.641 (0.93)	0.005	2.026 (0.87)	0.020
Less than high school	1	Ref	1	Ref	1	Ref
Mothers’ occupation						
Unemployed	−1.191 (1.15)	0.300	−1.159 (1.19)	0.332	−1.359 (1.12)	0.225
Worker	−2.413 (1.05)	0.023	−2.766 (1.10)	0.011	−2.939 (1.03)	0.004
Professional	1	Ref	1	Ref	1	Ref
Infant feeding method						
Breastfeeding	1.444 (1.36)	0.290	3.110 (1.40)	0.027	2.324 (1.32)	0.079
Formula	1	Ref	1	Ref	1	Ref
Home location						
Rural	−4.490 (1.04)	<0.001	−5.987 (1.08)	<0.001	−5.887 (1.01)	<0.001
Small Town	−3.744 (0.95)	<0.001	−6.042 (0.98)	<0.001	−5.476 (0.92)	<0.001
City	1	Ref	1	Ref	1	Ref

A manual model selection procedure was performed before the formal mixed model analysis. All variables listed in Table 1 were entered in the univariate mixed models separately and only those with a *p*-value < 0.20 were selected. Then all selected variables were entered in a mixed model and the one with the highest *p*-value was eliminated; this procedure was repeated until all *p*-values of type 3 tests of fixed effects stayed under 0.10 for the remaining variables. Abbreviation: Ref, Reference; VIQ, verbal IQ; PIQ, performance IQ; FIQ, full IQ.

**Table 3:** Association between categorical breakfast consumption frequency and IQ of the 2nd wave (*n* = 504).

Breakfast Variation	No. of Children (Total *n* = 504) ^$^	VIQ		PIQ		FIQ	
		Coefficient (SE)	*p*-Value	Coefficient (SE)	*p*-Value	Coefficient (SE)	*p*-Value
Univariable GLM							
More w1 + more w2	454 (90.1)	16.822 (5.10)	0.001	−0.632 (5.57)	0.910	10.570 (5.38)	0.050
Fewer w1 + more w2	24 (4.7)	11.808 (5.57)	0.035	−3.575 (6.09)	0.557	6.150 (5.88)	0.297
More w1 + fewer w2	21 (4.2)	11.695 (5.64)	0.039	−1.914 (6.16)	0.756	6.638 (5.96)	0.266
Fewer w1 + fewer w2	5 (1.0)	1	Ref	1	Ref	1	Ref
Multivariable GLM *							
More w1 + more w2	454 (90.1)	19.809 (5.46)	<0.001	0.304 (6.05)	0.960	12.947 (5.71)	0.024
Fewer w1 + more w2	24 (4.7)	17.662 (5.93)	0.003	0.330 (6.57)	0.960	11.986 (6.21)	0.054
More w1 + fewer w2	21 (4.2)	15.900 (6.01)	0.008	−0.900 (6.66)	0.893	9.872 (6.29)	0.117
Fewer w1 + fewer w2	5 (1.0)	1	ref	1	ref	1	ref

Abbreviation: more w1 + more w2: regular breakfast intake in wave1 + regular breakfast intake in wave2; fewer w1 + more w2: less regular breakfast intake in wave1 + regular breakfast intake in wave2; more w1 + fewer w2: regular breakfast intake in wave1 + less regular breakfast intake in wave2; fewer w1 + fewer w2: less regular breakfast intake in wave1 + less regular breakfast intake in wave2; * Adjusted for sex, parental education, mothers’ occupation, infant feeding method, and home location. ^$^ Numbers do not add to the sum of 511, because seven subjects completed questionnaires but failed to complete information about breakfast consumption. Abbreviation: GLM, general linear model; Ref, Reference; VIQ, verbal IQ; PIQ, performance IQ; FIQ, full IQ

**Table 4:** Dose-response relationship between breakfast consumption frequency and IQ scores/academic achievement of the 2nd wave (*n* = 835).

Breakfast Frequency *	VIQ		PIQ		FIQ		Academic Achievement	
	Coefficient (SE)	*p*-Value	Coefficient (SE)	*p*-Value	Coefficient (SE)	*p*-Value	Coefficient (SE)	*p*-Value
Univariate model I								
Breakfast frequency (numeric)	1.420 (0.369)	<0.001	0.383 (0.396)	0.334	1.125 (0.386)	0.004	0.126 (0.025)	<0.001
Univariate model II								
6–7 d/w	7.752 (2.544)	0.002	0.741 (2.721)	0.785	5.438 (2.655)	0.041	0.730 (0.179)	<0.001
3–5 d/w	4.952 (2.872)	0.085	−0.540 (3.072)	0.861	2.810 (2.996)	0.349	0.485 (0.199)	0.015
0–2 d/w		Ref		Ref		Ref		Ref
Multivariable model I ^$^								
Breakfast frequency (numeric)	1.131 (0.400)	0.005	0.180 (0.679)	0.904	0.829 (0.414)	0.046	0.134 (0.027)	<0.001
Multivariable model II ^$^								
6–7 d/w	6.760 (2.817)	0.017	0.062 (3.061)	0.984	4.437 (2.922)	0.130	0.831(0.195)	<0.001
3–5 d/w	5.307 (3.144)	0.092	0.222 (3.416)	0.948	3.422 (3.261)	0.295	0.575 (0.213)	0.007
0–2 d/w		Ref		Ref		Ref		Ref

* Breakfast consumption frequency was measured as a continuous variable and further categorized as 0–2, 3–5, 6–7 days per week. ^$^ All multivariable models adjusted for sex, fathers’ education, mothers’ education, mothers’ occupation, infant feeding method, and home location. Abbreviation: SE, standard error; VIQ, verbal IQ; PIQ, performance IQ; FIQ, full IQ; Ref, Reference

**Table 5:** Associations between IQ/academic achievement and food frequency intake at breakfast at age 12 in the 2nd wave (*n* = 835).

	VIQ		PIQ		FIQ		Academic Achievement	
	Adj Coef (SE)	*p*-Value	Adj Coef (SE)	*p*-Value	Adj Coef (SE)	*p*-Value	Adj Coef (SE)	*p*-Value
	Section I *							
Fruit/vegetables								
6–7 d/w	−0.409(1.302)	0.754	0.650(1.351)	0.631	0.232(1.301)	0.859	0.143(0.090)	0.111
3–5 d/w	−1.768(1.437)	0.219	−1.517(1.491)	0.309	−1.771(1.435)	0.218	0.012(0.098)	0.903
Grain/rice								
6–7 d/w	4.079(1.738)	0.019	4.129(1.829)	0.024	4.941(1.753)	0.005	0.273(0.130)	0.036
3–5 d/w	0.029(1.982)	0.988	0.721(2.087)	0.730	1.141(1.995)	0.568	0.043(0.144)	0.764
Meat/egg								
6–7 d/w	4.043(1.626)	0.013	3.781(1.694)	0.026	3.919(1.627)	0.016	0.301(0.110)	0.007
3–5 d/w	3.715(1.645)	0.024	2.153(1.714)	0.210	2.935(1.645)	0.075	0.235(0.112)	0.037
Dairy products								
6–7 d/w	1.736(1.505)	0.249	2.123(1.569)	0.177	2.102(1.504)	0.163	0.112(0.099)	0.260
3–5 d/w	1.499(1.586)	0.345	2.319(1.654)	0.162	2.296(1.584)	0.148	0.040(0.106)	0.705
Soy products								
6–7 d/w	2.609(1.413)	0.065	1.413(1.479)	0.340	2.681(1.413)	0.058	0.163(0.094)	0.085
3–5 d/w	2.362(1.184)	0.047	0.731(1.239)	0.556	1.863(1.185)	0.117	0.205(0.081)	0.012
	**Section II ^$^**							
Grain/rice								
6–7 d/w	3.562(1.768)	0.045	3.687(1.860)	0.048	4.559(1.767)	0.010	0.201(0.134)	0.133
3–5 d/w	−0.695(1.980)	0.726	0.165(2.083)	0.937	0.477(1.978)	0.809	−0.0001(0.146)	0.999
Meat/egg								
6–7 d/w	2.548(1.660)	0.126	2.406(1.746)	0.169	2.307(1.658)	0.165	0.232(0.114)	0.043
3–5 d/w	2.976(1.654)	0.073	1.277(1.739)	0.463	2.022(1.651)	0.221	0.192(0.114)	0.095

Reference groups used in GLM analysis: 0–2 d/w. * In section I, each breakfast type was entered separately into the different multivariable GLMs and its independent association with IQ scores and academic achievement was evaluated after controlling for covariates. ^$^ In section II, breakfast types that were statistically significant in models of section I were simultaneously selected into a new multivariable GLM adjusting for covariates.

**Table 6:** Effect of IQ scores and breakfast consumption frequency on academic achievement in the 2nd wave (*n* = 35).

	Model I		Model II	
	Coefficient (SE)	*p*-Value	Coefficient (SE)	*p*-Value
	Models: AA = VIQ + breakfast frequency			
Breakfast frequency				
6–7 d/w	0.822 (0.212)	<0.001	0.951 (0.244)	<0.001
3–5 d/w	0.766 (0.236)	0.001	0.822 (0.268)	0.002
0–2 d/w		Ref		Ref
VIQ	0.021 (0.003)	<0.001	0.022 (0.004)	<0.001
	Models: AA = PIQ + breakfast			
Breakfast frequency				
6–7 d/w	0.988 (0.215)	<0.001	1.099 (0.248)	<0.001
3–5 d/w	0.893 (0.241)	<0.001	0.947 (0.274)	0.001
0–2 d/w		Ref		Ref
PIQ	0.008 (0.003)	0.014	0.011 (0.004)	0.002
	Models: AA = FIQ + breakfast			
Breakfast frequency				
6–7 d/w	0.890 (0.213)	<0.001	1.004 (0.245)	<0.001
3–5 d/w	0.825 (0.238)	0.001	0.868 (0.269)	0.001
0–2 d/w		Ref		Ref
FIQ	0.017 (0.003)	<0.001	0.019 (0.004)	<0.001

Model I: academic achievement (AA) = IQ + breakfast frequency. Model II: academic achievement (AA) = IQ + breakfast frequency + adjusted covariates. Abbreviation: SE, standard error; VIQ, verbal IQ; PIQ, performance IQ; FIQ, full IQ.

## Data Availability

The data that support the findings of this study are available from the corresponding author J.L. upon reasonable request.

## Supplementary Materials

The following is available online at https://www.mdpi.com/article/10.3390/nu13062080/s1, Table S1: Crude associations of IQ scores/academic achievement with baseline characteristics.
