# Supplementary material for: Breakfast Consumption Habits at Age 6 and Cognitive Ability at Age 12: A Longitudinal Cohort Study
Source: Nutrients. 2021 Jun 17;13(6):2080. doi: 10.3390/nu13062080 (PMC8234310; doi:10.3390/nu13062080)
Supplement: Supplementary file 1 [file nutrients-13-02080-s001.zip › nutrients-1234883-supplementary.pdf]

Table S1. Crude associations of IQ scores/academic achievement with baseline characteristics

	VIQ		PIQ		FIQ		Academic achievement	
	**raw coef (SE)**	**P-value**	**raw coef (SE)**	**P-value**	**raw coef (SE)**	**P-value**	**raw coef (SE)**	**P-value**
Sex (male)	1.878 (0.919)	0.041	3.926 (0.910)	<0.001	3.253 (0.913)	<0.001	-0.378 (0.067)	<0.001
Fathers' education								
High school	3.970 (1.072)	<0.001	2.390 (1.098)	0.030	3.890 (1.067)	<0.001	0.033 (0.083)	0.685
College or higher	8.951 (1.105)	<0.001	5.488 (1.133)	<0.001	8.565 (1.102)	<0.001	0.313 (0.084)	<0.001
Mothers' education								
High school	5.506 (1.030)	<0.001	3.599 (1.062)	<0.001	5.511 (1.030)	<0.001	0.028 (0.078)	0.721
College or higher	9.192 (1.193)	<0.001	4.907 (1.230)	<0.001	8.375 (1.193)	<0.001	0.330 (0.089)	<0.001
Fathers' occupation								
Unemployed	-6.611 (2.275)	0.004	-4.800 (2.255)	0.034	-6.843 (2.239)	0.002	-0.283 (0.198)	0.153
Labor Worker	-5.170 (0.981)	<0.001	-3.823 (0.972)	<0.001	-5.337 (0.966)	<0.001	-0.125 (0.072)	0.085
Mother's occupation								
Unemployed	-5.713 (1.242)	<0.001	-4.031 (1.240)	0.001	-5.813 (1.222)	<0.001	-0.144 (0.092)	0.117
Worker	-6.076 (1.117)	<0.001	-5.024 (1.116)	<0.001	-6.512 (1.101)	<0.001	-0.204 (0.081)	0.012
Parents divorced or separated (no)	1.963 (2.824)	0.487	-1.206 (2.861)	0.673	1.161 (2.913)	0.691	0.215 (0.217)	0.322
Maternal age at childbirth	0.236 (0.174)	0.175	0.277 (0.172)	0.108	0.330 (0.173)	0.057	-0.001 (0.012)	0.935
Infant feeding method								
Breastfeeding	3.473 (2.009)	0.084	3.152 (2.017)	0.119	3.722 (2.004)	0.064	0.296 (0.139)	0.033
Breastfeeding duration	-0.023 (0.157)	0.884	-0.261 (0.158)	0.099	-0.139 (0.157)	0.376	-0.029 (0.012)	0.013
Home location								
Rural	-4.306 (1.438)	0.003	-4.531 (1.426)	0.002	-5.415 (1.424)	<0.001	-0.165 (0.105)	0.117
Small Town	-3.631 (1.208)	0.003	-4.163 (1.198)	<0.001	-4.655 (1.190)	<0.001	-0.067 (0.095)	0.481
Living space per person (m2)	-0.024 (0.035)	0.488	-0.043 (0.035)	0.214	-0.036 (0.035)	0.296	-0.001 (0.003)	0.785
Siblings								
Have at least one sibling	-5.418 (1.217)	<0.001	-1.054 (1.211)	0.385	-3.664 (1.211)	0.003	-0.272 (0.090)	0.003

Reference groups used in GLM analysis were: female (gender), less than high school (education), professional (occupation), yes (parent's divorce or separation), formula (feed type during infancy), city (home location), and no siblings (siblings).
